# Supplementary figures and images for: Epidemiology and risk factors of Japanese encephalitis in Taiwan, 2010–2022
Source: PLoS Negl Trop Dis. 2023 Oct 2;17(10):e0011421. doi: 10.1371/journal.pntd.0011421 (PMC10569588; doi:10.1371/journal.pntd.0011421)

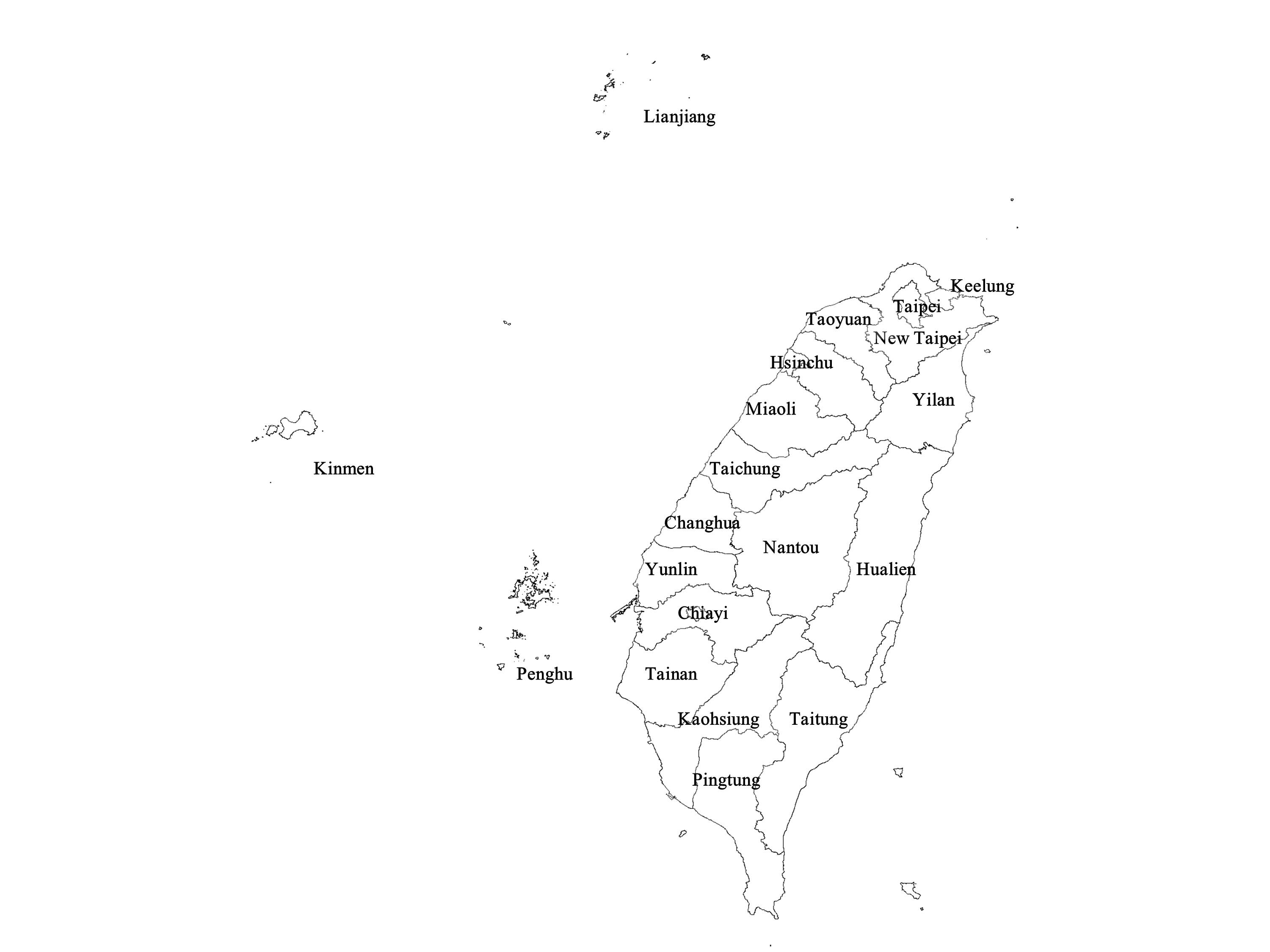

Supplement: S1 Fig — was generated using Statistical Analysis Software version 9.4 (SAS Institute, Cary, NC, USA). The link to the base layer of the map is: https://data.gov.tw/dataset/7442. (TIF) [file pntd.0011421.s001.tif]
