# Supplementary material for: Epidemiology and risk factors of Japanese encephalitis in Taiwan, 2010–2022
Source: PLoS Negl Trop Dis. 2023 Oct 2;17(10):e0011421. doi: 10.1371/journal.pntd.0011421 (PMC10569588; doi:10.1371/journal.pntd.0011421)
Supplement: Attachment — Submitted filename: review_PLoSNTD_JE.pdf [file pntd.0011421.s003.pdf]

Title: Epidemiology and risk factors of Japanese encephalitis in Taiwan, 2010-2022

This is a very well-written manuscript about Japanese encephalitis (JE) in Taiwan.

It's a 13-year retrospective epidemiological study from 2010 to 2022 that documented 313 cases of JE.

Several risk factors were studied, including gender, age, date of birth, immunization protocols, region and city of residence, and occupation. The authors suggest a new vaccination protocol that could improve the level of immunization of Taiwanese population at risk of JE.

However, here are a few comments to consider:

Line 105-106: … the knowledge may… are prevalent. The sentence is unclear; please rephrase.

Line 117-118: the Centers for Diseases Control ... diagnosis of JE. Please add a reference.

Line 122: …or tissue ~~to the CDC~~..: Please delete to the CDC: repetition.

Line 160: … for JE was developed… in 1998: please add a reference.

Line 164: through real time polymerase chain reaction: please add a reference.

Line 174: Please specify the source of your mortality data.

Line 225: Please indicate in materials and methods, your method for estimating the fatality rate, and explain why you used 17 annual deaths which you divided by 313 cases gathered over 13 years. Why didn't you use the 22 average annual cases?

Line 281-282: Please verify the value of Taiwan's one-year case fatality rate.

Question: aren't there problems of under-reporting in Taiwan? what about cases that don't show up for medical consultations?

Line 313: seropositive rate: post vaccination or post natural infection? Can you distinguish between the two?
