# Supplementary material for: Epidemiology and risk factors of Japanese encephalitis in Taiwan, 2010–2022
Source: PLoS Negl Trop Dis. 2023 Oct 2;17(10):e0011421. doi: 10.1371/journal.pntd.0011421 (PMC10569588; doi:10.1371/journal.pntd.0011421)
Supplement: Attachment — Submitted filename: Reply20230803.docx [file pntd.0011421.s004.docx]

Reply to “Epidemiology and risk factors of Japanese encephalitis in Taiwan, 2010–2022” (PNTD-D-23-00664) - [EMID: f11e0e9eff502388]

Dear Dr. Parker and Dr. Victoria Brookes,

Thank you for your letter and arrangements. We are honored and delighted to receive your attention and have the opportunity to make revisions based on the reviewers' suggestions. Below, we have provided a point-by-point response to the comments made by both reviewers. Additionally, we have uploaded two versions of the revised manuscript: one with tracked changes and the other a clean version.

Sincerely yours,

Jen-Yu Hsu

Medical Officer

Centers for Disease Control, Ministry of Health and Welfare, Taipei, Taiwan

**Reviewer #1:**

**Reviewer’s comment:**

Methods

Clear descriptions of the aims, demographic variables, and datasets, as well as the diagnostic methods considered for confirming a case of JEV infection were provided.

The stratification used to calculate relative risks is useful, but without any sort of confounding analysis.

**Response:**

Thank you very much for the kind comment. We also appreciate the reviewer's time and assistance in improving the quality of our work. We fully agree that conducting an analysis to control for confounding factors is essential. However, limited by the availability of records of patients with confirmed Japanese encephalitis and the lack of complete records of those with unconfirmed Japanese encephalitis, we had to perform separate analyses for each risk factor using various datasets. We have included this limitation in detail in the "Limitation" section and hope that in the future, efforts can be made to improve the integration of various reporting systems in Taiwan to facilitate the execution of multivariable analysis for controlling confounding factors (Lines 359–360, Page 23).

**Reviewer’s comment:**

Results

Overall, the result section is well-developed; although it may lead to some confusion to only have the risk factor analysis at the end of the section, when the comparisons are made earlier on. The figures and the tables are suitable.

**Response:**

Thank you for the kind reminder. We have decided to break down "The risk factor analysis" at the end of the “Results” section and describe its content separately within each risk factor paragraph. This approach will help avoid confusion (Lines 242–249, Page 16; Lines 263–271, Pages 17–18).

**Reviewer’s comment:**

The conclusions drawn from the data are well-supported, and the paper effectively addresses most of its limitations. It provides a thorough explanation of the potential reasons behind the observed differences in risk groups. A notable limitation arises from solely stratifying the data based on one variable of interest, such as sex or profession, without implementing a risk factor analysis to adjust relative risks (RRs) for potential confounding factors (by multiple stratification or regression).

**Response:**

Thank you for pointing out this limitation. The inability to use multiple regression to control for confounding factors indeed poses a significant constraint on obtaining more precise estimations of relative risks. Following your suggestion, we perform multiple stratifications based on gender and each risk factor, and we have included the results in the "Results" section (Lines 242–249, Page 16; Lines 263–271, Pages 17–18). However, due to the data's nature, further stratified analyses are not feasible.

**Reviewer’s comment:**

Editorial and Data Presentation Modifications?

1) The paper could benefit of controlling for confounding, perhaps through multiple stratification, building upon the stratification performed in the paper, to simultaneously adjust for several of the aforementioned risk factors. For instance, the higher risk of JE in males may be attributed to their involvement in high-risk professions, increased participation in outdoor activities (as discussed in the paper's discussion section), lower vaccination coverage... Otherwise, it could be mentioned in the discussion some literature in Taiwan that conducted similar analysis with confounding.

**Response:**

Regarding JE, there are currently no studies in Taiwan that discuss confounding analysis. Unfortunately, we cannot utilize multiple regression to further analyze the reasons why males might be more susceptible to JE compared to females in this study. However, through stratified analysis, we found that in cohort 1, females had a higher relative risk than males compared to cohort 3. This finding may be related to the fact that, in the early days, males were more prone to natural infections and developed antibodies in Taiwan, which could indirectly indicate that Taiwanese males had more outdoor exposure experiences than females. Additionally, by including a description of the majority of male workers in the agricultural and forestry industries, we can supplement the explanation that males were more likely to be exposed to outdoor activities (Lines 309–313, Pages 20–21).

**Reviewer’s comment:**

2) Consider clarifying that “herd immunity may not necessarily work” (line 81) due to the zoonotic nature of the virus (so immunizing humans would not eliminate the virus from the region). Citation: https://www.ncbi.nlm.nih.gov/books/NBK219063/

**Response:**

Thank you for your guidance. We have revised the statement as follow: "As JEV is transmitted in a zoonotic cycle, with humans being incidental hosts, a vaccination program would not confer herd immunity. Disease reduction depends on individual vaccination and the adoption of measures to avoid mosquito bites." This clarification has been incorporated along with the suggested citation (Lines 79–81, Page 6).

**Reviewer’s comment:**

3) In the abstract, should “population” in line 43 be “population per year” or “person-years”?

**Response:**

Thank you for pointing out our oversight. We have now updated the terms "population" to "population per year" in the Abstract and Author Summary (Line 41, Page 3; Lines 63–64, Page 5).

**Reviewer’s comment:**

4) In line 92, it is mentioned that the majority of Japanese encephalitis patients in Taiwan during the 2000s were unvaccinated individuals. Additionally, in lines 276-278, it is stated that the vaccination rate has been high since the 1960s. Hence, when discussing the younger population (born after 1962), as the recommendation of the paper is to provide vaccination, it could be beneficial to mention some existing literature and available data on the reasons behind vaccination rates not reaching 100% in Taiwan and where research should move to. This exploration may shed light on potential factors contributing to higher incidence rates in men, beyond differences in outdoor activities mentioned in lines 300-1 (but without mentioning other studies with similar results).

**Response:**

In Taiwan, the majority of JE cases occur in individuals who have not undergone vaccinations or have not completed the vaccination schedule (born before 1976). For those born after 1976, over 95% have undergone complete vaccinations and would be less susceptible to JE. We recommend that individuals with incomplete vaccination or uncertain vaccination history should consider completing the vaccination schedule. However, in Taiwan, the incidence and mortality rates of JE are relatively low; therefore, at this time, the government does not plan to implement widespread government-subsidized or mandatory vaccinations. Instead, serological antibody surveys could be considered in the future. Additionally, we have included relevant literature and the results of stratified analysis to suggest that Taiwanese males may have a higher likelihood of engaging in outdoor activities or having exposure histories compared to females (Lines 309–313, Pages 20–21).

**Reviewer’s comment:**

5) Is there any available information regarding the vaccination status of individuals in Cohorts 2 and 3, considering that vaccination rates were high but not ~100%? Only in the abstract it is mentioned that vaccination persons "without vaccination" or "unknown vaccination status", but the lack of data on individual vaccination status of cases could perhaps be best clarified as a limitation.

**Response:**

Unfortunately, we cannot precisely determine individuals' vaccination status, so we have to rely on their birth year for estimation. As you mentioned, the lack of vaccination records is a limitation, and we have described it as the second limitation (Lines 352–353, Page 23).

**Reviewer’s comment:**

Summary and General Comments

The article by Hsu et al. is clear and the writing style is fluid and easily understandable. The article sheds light on an important regional topic, aiming to enhance the understanding of the epidemiology of Japanese encephalitis. It delves into various aspects including the clinical manifestations, high-risk groups, vaccine history/policy and seasonality of JE in Taiwan.

**Response:**

Thank you for your thorough understanding of this article and for providing many constructive suggestions.

**Reviewer #2:**

**Reviewer’s comment:**

This is a very well-written manuscript about Japanese encephalitis (JE) in Taiwan.

The objectives were clearly specified. It's a 13-year retrospective epidemiological study from 2010 to 2022 that documented 313 cases of JE.

Several risk factors were studied, including gender, age, date of birth, immunization protocols, region and city of residence, and occupation.

The method for calculating the fatality rate must be verified.

**Response:**

Thank you for taking the time to read our article and provide valuable insights.

**Reviewer’s comment:**

Line 105-106: … the knowledge may… are prevalent. The sentence is unclear; please rephrase.

**Response:**

Apologies for the previous lack of clarity. It has been revised as follow: "While mosquito-borne diseases have been acknowledged as occupational hazards for individuals traveling to endemic areas, they may not be officially recognized as such in regions where these diseases are prevalent." (Lines 102–104, Page 7).

**Reviewer’s comment:**

Line 117-118: the Centers for Diseases Control ... diagnosis of JE. Please add a reference.

**Response:**

Thank you for your suggestion. We have added two references to our manuscript: "The incidence of Japanese encephalitis in Taiwan--a population-based study" by Hsu et al. (PLoS Negl Trop Dis. 2014;8(7):e3030) and "The epidemiology of Japanese encephalitis on Taiwan during 1966-1997" by Wu et al. (Am J Trop Med Hyg. 1999;61(1):78-84) (Lines 114–116, Page 9).

**Reviewer’s comment:**

Line 122: …or tissue to the CDC..: Please delete to the CDC: repetition.

**Response:**

Thank you for pointing out our oversight. Deleting this redundant word has made the sentence much smoother (Line 120, Page 9).

**Reviewer’s comment:**

Line 160: … for JE was developed… in 1998: please add a reference.

**Response:**

We have included the reference by Litzba et al. entitled "Evaluation of serological diagnostic test systems assessing the immune response to Japanese encephalitis vaccination" (PLoS Negl Trop Dis. 2010;4(11):e883) (Line 158, Page 11).

**Reviewer’s comment:**

Line 164: through real time polymerase chain reaction: please add a reference.

**Response:**

Thank you. We have included the reference by Moureau et al. entitled "A real-time RT-PCR method for the universal detection and identification of flaviviruses" (Vector Borne Zoonotic Dis. 2007;7(4):467-77) (Line 162, Page 11).

**Reviewer’s comment:**

Line 174: Please specify the source of your mortality data.

**Response:**

Thank you for your reminder. We specify the source of mortality data as follows: "Mortality data from the National Health Insurance Research Database, which encompasses around 99.9% of Taiwan's population, was obtained through mandatory reporting to the NIDRS." Additionally, we extended the observation period from May 29, 2023, to August 1, 2023 (Lines 172–176, Page 12).

**Reviewer’s comment:**

Line 225: Please indicate in materials and methods, your method for estimating the fatality rate, and explain why you used 17 annual deaths which you divided by 313 cases gathered over 13 years. Why didn’t you use the 22 average annual cases?

Line 281-282: Please verify the value of Taiwan's one-year case fatality rate.

**Response:**

We apologize for not describing it clearly. The incidence and mortality rates of JE were calculated by dividing the total number of occurrences during the follow-up period by the total person-years of follow-up. The case fatality rate was calculated by dividing the number of deaths from the disease by the total number of confirmed cases (Lines 198–201, Page 13). Out of the 313 patients with confirmed JE, 17 deaths occurred within one year of diagnosis, resulting in a case fatality rate of 5.4% (Lines 225–226, Page 15).

**Reviewer’s comment:**

Question: aren't there problems of under-reporting in Taiwan? what about cases that don't show up for medical consultations?

**Response:**

In Taiwan, seeking medical attention is relatively easy with the wide coverage of the National Health Insurance and the distribution of health care facilities around the island. However, indeed, some people may not show up for medical consultations, leading to lower numbers of confirmed cases. Between 2010 and 2022, attending physicians reported a total of 3,927 cases of suspected Japanese encephalitis, out of which 313 were confirmed as Japanese encephalitis. Under-reporting is of course a concern for us, so we have listed this as the first limitation of our study, and revision have been made to the text accordingly (Lines 349–351, Page 23).

**Reviewer’s comment:**

Line 313: seropositive rate: post vaccination or post natural infection? Can you distinguish between the two?

**Response:**

Thank you for your thorough review. Indeed, we cannot distinguish whether the seropositivity was due to previous vaccination or natural infection. However, given the higher incidence rate in the eastern region coupled with the lower seropositive rate, we speculate that this was likely a result of post-vaccination (Lines 324–327, Page 21).

**Reviewer’s comment:**

The authors suggest a new vaccination protocol that could improve the level of immunization of Taiwanese population at risk of JE.

**Response:**

Thank you for the comment. In the future, more research will be needed to prove the effectiveness of the new vaccination protocol.
